# The use of point-of-care ultrasound in nurses’ clinical practice as a foundation for patient safety

**DOI:** 10.1590/0034-7167.202477suppl0201

**Published:** 2024-03-11

**Authors:** Vinicius Batista Santos, Wesley Pinto da Silva, Mónica Francisca Santana Apablaza, Thiago Vital da Silva, Fernanda Raphael Escobar Gimenes

**Affiliations:** I Universidade Federal de São Paulo, Escola Paulista de Enfermagem. Member of the Point-of-Care Ultrasound Research Study Center and the Ultrasound Academy for Nurses. São Paulo, São Paulo, Brazil; II Faculdade EnsinE and Centro de Treinamento em Emergências do Sul Fluminense. Founding Member of the Ultrasound Academy for Nurses and Member of the Point-of-Care Ultrasound Research Study Center. Rio de Janeiro, Rio de Janeiro, Brazil; III Universidade de São Paulo, Escola de Enfermagem de Ribeirão Preto, Department of General and Specialized Nursing. São Paulo, Ribeirão Preto, Brazil

Recently, another case of serious harm associated with healthcare was reported by the media. A young pregnant woman had her hand and wrist amputated after giving birth to her third child in a hospital in Rio de Janeiro^(1)^. After the incident caused by vascular access, the woman suffered hemorrhage and was readmitted.

The harm caused to pregnant women as well as to other patients can be avoided with the use of bedside ultrasound. Nurses with advanced knowledge and skills to manage point-of-care ultrasound (POCUS) have a powerful semiological tool to reduce the risks of serious and potentially fatal harm related to healthcare. In the field of nursing, POCUS is used in diverse populations and at different levels of healthcare – from primary care to critical and emergency care.

The initial use of POCUS by nurses was for invasive procedures, such as peripheral venous puncture, insertion of central and arterial catheters, showing an improvement in the accuracy of these interventions^(2)^. Subsequently, research explored its effectiveness in assessing bladder residual volume, confirming bladder catheter passage, assessing gastric residual volume to prevent bronchopulmonary aspiration, confirmation of feeding tube passage and arteriovenous fistula flow and patency assessment in patients undergoing dialysis therapy^(3)^.

For these reasons, insonation is being introduced into clinical practice as the fifth element of physical examination, which traditionally includes inspection, palpation, percussion and auscultation. Furthermore, ultrasound can be used by nurses to complement the physical examination, assist in clinical reasoning, support the identification of nursing diagnoses as well as monitor indicators of results sensitive to nursing interventions.

Among the main studies that used POCUS as a complement to physical examination, optic nerve sheath diameter measurement, pulmonary assessment with a focus on identifying complaints of dyspnea, cardiac and inferior vena cava assessment to measure volume status, assessment in patients victims of multisystem trauma for early identification of signs of clinical severity such as bleeding and assessment of fecal impaction in those patients with impaired intestinal elimination stand out. More recently, studies were identified by nurses to assess pressure injuries using POCUS^(3)^.

In Brazil, in 2021, through Resolution 679^(4)^, the Federal Nursing Council authorized nurses to perform ultrasound at the bedside and in pre-hospital environments for non-nosological purposes, aiming to guide procedures and identify phenomena treatable by nursing.

These advances led to the creation in 2022 of the Ultrasound Academy for Nurses, aimed at bringing together nurses and researchers in the field of ultrasound to share experiences and improve knowledge. Subsequently, in February 2023, the first Center for Research and Development in Point-of- Care Ultrasonography for nurses was created, linked to the Brazilian National Council for Scientific and Technological Development (CNPq - *Conselho Nacional de Desenvolvimento Científico e Tecnológico*), with the mission of encouraging research with POCUS.

However, the use of this technology still faces challenges in nursing, especially in proving its impact on patient safety and as a clinical indicator for identifying nursing diagnoses and assessing results sensitive to nursing interventions. Therefore, researchers are invited to advance in this field to better understand the potential of POCUS in advanced nursing practice.
